# Evaluation of Adipose Cell-Based Therapies for the Treatment of Thumb Carpometacarpal Joint Osteoarthritis

**DOI:** 10.3390/biom12030473

**Published:** 2022-03-20

**Authors:** Eleni Karagergou, Theodora Ligomenou, Byron Chalidis, Dimitrios Kitridis, Sophia Papadopoulou, Panagiotis Givissis

**Affiliations:** 1 Department of Burns, Plastic Surgery and Hand Surgery, Georgios Papanikolaou Hospital, 57010 Thessaloniki, Greece; t.ligomenou@gmail.com (T.L.); sophipap@hotmail.com (S.P.); 2 1st Orthopaedic Department, School of Medicine, Georgios Papanikolaou Hospital, Aristotle University of Thessaloniki, 57010 Thessaloniki, Greece; byronchalidis@gmail.com (B.C.); dkitridis@gmail.com (D.K.); pgivissis@gmail.com (P.G.)

**Keywords:** fat grafting, fat transfer, carpometacarpal arthritis, thumb arthritis

## Abstract

Adipose tissue and its regenerative products which are isolated with enzymatic or mechanical processing of the harvested fat have been studied in a wide range of degenerative diseases, including osteoarthritis of the knee and hip. Intra-articular injection of these products can provide symptomatic relief of pain and postpone surgery. However, their use in the treatment of thumb carpometacarpal joint (CMCJ) osteoarthritis is limited and just a few studies have been published on that topic. For this reason, a review of the literature was performed by a thorough search of eight terms using the Pubmed database. In total, seven human studies met the selection criteria, including case-control studies, case-series and one case report. In all studies, intra-articular injection of autologous fat in osteoarthritic thumb CMCJ provided reduction in pain and improvement in hand function. Grip and pinch strength showed variable results, from no change to significant improvement. Fat-processing techniques were based on centrifugation and mechanical homogenization but biological characterization of the injected cells was not performed in any study. Although the results are encouraging, a uniformly standardized method of fat processing and the conduction of randomized controlled trials in the future could better evaluate the effectiveness of this procedure for thumb CMCJ osteoarthritis.

## 1. Introduction

The presence of mesenchymal stem cells in adipose tissue was first described in 2001 by Zuk et al. [1]. These cells have been characterized by strong regenerative properties and a multilineage differentiation potential comparable to bone marrow mesenchymal stem cells [1,2,3,4]. Since then, the regenerative capacity of adipose tissue along with its anti-inflammatory and immunomodulatory properties have been a constant field of research and clinical application. From pure macrofat and nanofat grafting till the enzymatically isolated adipose derived stromal vascular fraction (SVF) and cultured stem cells, the adipose tissue has been studied in a wide range of degenerative diseases [5].

In particular, adipose-derived SVF is a popular product that has been clinically applied to joints with osteoarthritic changes [6,7,8,9]. It is isolated after enzymatic digestion and centrifugation of the harvested fat and consists of a heterogenous cell population of mesenchymal stem cells, pericytes, endothelial progenitor cells and immune cells, with strong regenerative capacity and a potential synergistic effect on immunomodulation, inflammation and angiogenesis [10]. Intra-articular injection of autologous SVF derived from lipoaspirate has been used for the symptomatic treatment of grade 3 and 4 knee osteoarthritis, with a significant clinical and radiological improvement at one year of follow-up [6,7]. In a large prospective and multicenter case control study which included a total of 1128 patients, intra-articular SVF injection was performed in patients with grade 2 to 4 osteoarthritis of large weight bearing joints (knee and hip) or other joints (shoulder, elbow, wrist, ankle and foot). Patient-reported symptoms were improved in 91% of patients 12 months after SVF cell therapy [8]. In a phase I/II clinical trial, intra-articular injection of high dose (1.0 × 10^8^ cells) precultured adipose derived stem cells in osteoarthritic knees improved joint function and pain and also led to cartilage regeneration [9].

Despite the recent popularity of the application of fat tissue in the symptomatic treatment of knee and hip osteoarthritis, its high abundance within the body and its high surgical accessibility, there are limited data regarding its use in small joint arthritis.

Thumb carpometacarpal joint (CMCJ) arthritis is the second most common location for degenerative osteoarthritis in the wrist and hand and several surgical treatments have been proposed so far, from arthrodesis to motion sparing procedures—such as trapeziectomy alone or in combination with interposition of autograft, allograft and ligamentoplasty [11,12,13]. These techniques are mainly indicated for the treatment of the late stages of the disease (stage 3 and 4 according to Eaton classification) [14].

However, there is a great variety in surgeons’ preferences and none of the available techniques show superiority over another [15]. Consequently, a less invasive procedure that could provide symptomatic relief or postpone surgery seems desirable. Given the popularity of fat and its regenerative cellular products, the present study was conducted in order to provide a thorough analysis of their current clinical use in the treatment of thumb carpometacarpal joint (CMCJ) arthritis as well as to establish this technique as regenerative treatment option for thumb CMCJ and other small joint arthritis.

## 2. Materials and Methods

A comprehensive search was conducted by two independent reviewers in the advanced search of the PubMed database using the following terms: (“trapeziometacarpal joint osteoarthritis” OR “first carpometacarpal joint osteoarthritis” OR “thumb osteoarthritis”) AND (“fat grafting” OR “stromal vascular fraction” OR “nanofat” OR “microfat” OR “adipose-derived stem cells”). Inclusion criteria were the intra-articular use of fat or adipose-derived regenerative cell therapy for thumb CMCJ arthritis and the subsequent reported treatment effect in pain, strength and hand function. Studies included were studies reporting clinical outcomes. Exclusion criteria were articles reporting fat grafting in other joints or treatment of thumb CMCJ arthritis without intra-articular injection of fat or adipose regenerative cells or combination of fat grafting with other techniques.

The primary outcome was the effect of fat and its regenerative cells in hand pain and function; all other outcomes were considered secondary. The information extracted from each article included: treatment indication and stage of osteoarthritis, processing technique of fat and volume injected, whether the injection was performed under fluoroscopic guidance, grip and pinch strength values, complications and radiographic improvement on follow-up. A formal statistical analysis of the included studies was not performed because of the heterogeneity in methodology. Instead, a systematic review of the reported outcomes of each study was conducted to evaluate the current evidence regarding the efficacy of the technique.

## 3. Results

**Figure 1:**
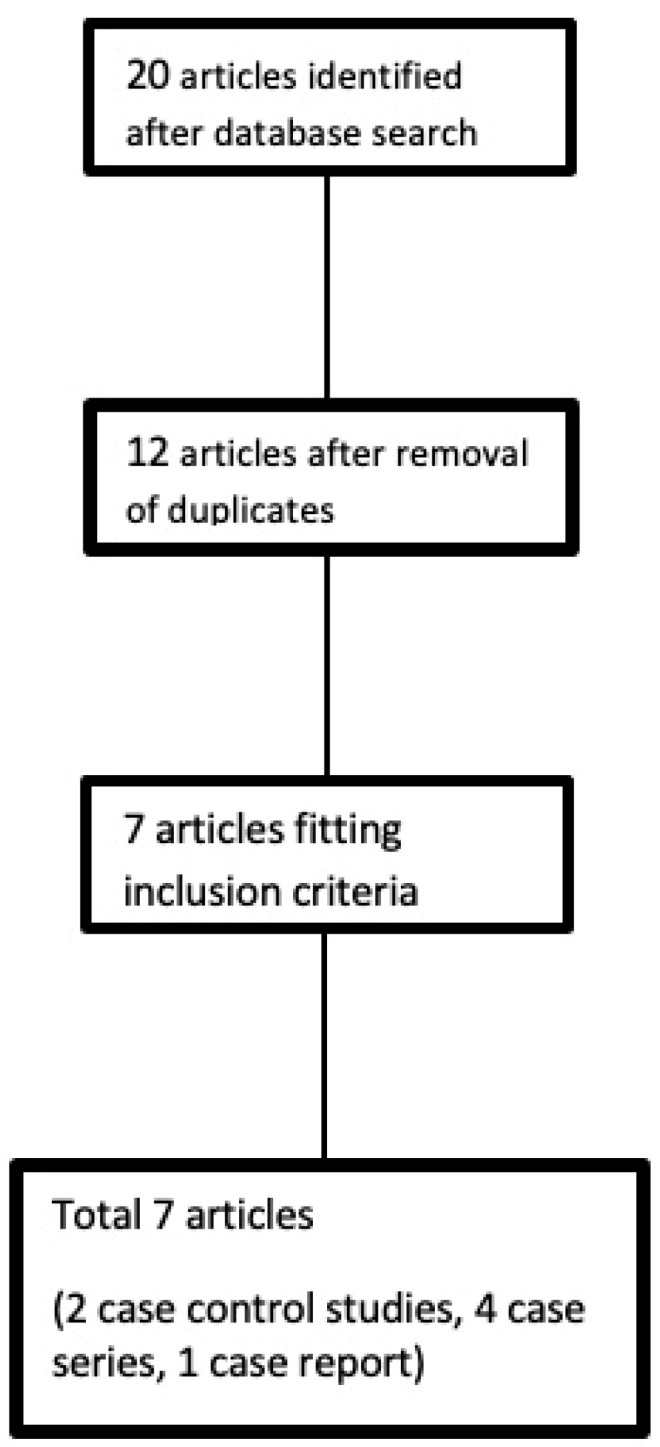
Flow diagram of the search and selection strategy of included articles.

The primary search yielded 20 articles. After the removal of duplicates, 12 of these were found to be distinct. Two articles that met the inclusion criteria were in German and were translated, while the rest were in English. Five more studies were excluded after reviewing their full text, because they did not meet the inclusion criteria. In one of them, fat grafting to thumb CMCJ was performed after arthroscopic synovectomy; this study was excluded because it was not possible to evaluate the effectiveness of fat injection as single procedure [16]. Thus, we reviewed a total of seven articles (Figure 1 and Table 1).

These articles were based on human studies, including case control studies (*n* = 2), case series (*n* = 4) and a case report (*n* = 1). There were no randomized controlled trials. In all studies, after liposuction, there was a minimal processing of the harvested adipose tissue either by centrifugation (*n* = 4) or by mechanical homogenization alone *(n* = 1) or in combination with filtration (*n* = 1) and decantation (*n* = 1). None of the studies described extraction of the regenerative cell fraction by enzymatic or mechanical method, nor examination of the injected tissue for cell count or viability. The majority of studies included patients that had failed conservative treatment with splinting, physical therapy and anti-inflammatory medication [17,18,19,20]. In all studies, intra-articular injection of 1 to 2 mL of fat into the thumb carpometacarpal joint was performed under fluoroscopic X-ray guidance. In all studies, pre- and post-treatment pain was evaluated according to the Visual Analogue Scale (VAS) but two different scales were used (1–10 or 1–100), making any statistical analysis or graphics not possible. A different methodology was used for measurement of pinch and grip strength.

**Table 1:** Summary of findings of reviewed articles.

Author	Type of Study	Number of Patients	Eaton Stage	Fat Technique	Fat Volume	VAS	Pinch & Grip	DASH or MHQ	Follow Up	Study Weaknesses
Haas et al., 2017 [21]	Case control	24 patients 12 fat grafting 12 triamcinolone	I to III	Mechanical homogenization	1–1.5 Ml	Significant reduction after fat grafting	No significant improvement within/ between groups	Significant improvement after fat grafting	3 months	No randomization Short follow-up Small sample size
Erne et al., 2018 [22]	Case control	21 patients 12 control (Lundborg arthroplasty) 9 (fat grafting)	III & IV	Centrifugation (Coleman )	1.3 mL	Improvement but no significant difference between groups	Improvement but no significant difference between groups	Improvement but no significant difference between groups	12 months	No randomization Small sample size
Herold et al., 2014 [23]	Case series	5 patients	II & III	Centrifugation (Coleman )	1.5 mL	Reduction but without statistical significance	Improvement but without statistical significance	Significant improvement after fat grafting	3 months	Lack of controls Small sample size Short follow-up
Herold et al., 2017 [17]	Case series	50 patients	II, III & IV	Centrifugation (Coleman )	1 mL	Significant reduction after fat grafting	Significant improvement after fat grafting	Significant improvement after fat grafting	12 months	Lack of controls
Haas et al., 2019 [18]	Case series	99 patients	I to III	Filtration & mechanical homogenization	1–2 mL	Significant reduction after fat grafting	No significant improvement after fat grafting	Significant improvement after fat grafting	12 months	Lack of controls
Froschauer et al., 2020 [19]	Case series	31 patients	II & III	Decantation/ mechanical homogenization	1 mL	Significant reduction after fat grafting	No significant improvement after fat grafting	Significant improvement after fat grafting	2 years	Lack of controls
Bohr et al., 2015 [20]	Case report	1 patient	II	Washing/centrifugation (Coleman)	1 mL	Not studied	Not studied	Improvement after fat grafting	12 months	Small sample size, lack of clinical evaluation

This review revealed only two case control studies comparing intra-articular fat injection with corticosteroid injection or surgery [21,22]. Haas et al. [21] described the outcome of autologous fat transplantation or corticosteroid injection in patients suffering from stage I to III thumb CMCJ arthritis. The fat-processing technique was based on mechanical homogenization using two Luer lock syringes, but no further information regarding the type of the injected product was mentioned (e.g., macro-, micro- or nanofat). After a short follow-up of three months, the fat group showed a significant reduction in pain and improvement in quality of life by means of QuickDASH and the Michigan Hand Questionnaire (MHQ), while the corticosteroids group, although it initially demonstrated an improvement, fell below the preoperative level after six weeks. However, both methods had no effect in grip strength and the follow-up period was too short. In the second case control study, Erne et al. [22] treated 21 patients with symptomatic and advanced thumb CMCJ arthritis (Eaton stages III and IV) with either autologous fat grafting (*n* = 9 patients) or with Lundborg resection arthroplasty (*n* = 12 patients). Allocation to each group was based on patients’ preference for a less invasive or a traditional surgical procedure. The time until complete resolution of pain was significantly shorter in the fat group (1.7 months compared with 5.7 months in the resection group). Pain according to VAS scale, pinch and grip strength, and DASH score showed comparable improvement in both groups, after 12 months. The authors also concluded that the duration of the procedure was significantly shorter in the fat group compared to the resection arthroplasty group. Although this is the only study that compared a less invasive procedure, such as fat grafting, with a traditional surgical technique for thumb CMCJ arthritis, it is characterized by a small sample size, lacks randomization and therefore is prone to selection bias.

In a small case series of five patients, Herold et al. [23] injected autologous fat in the thumb CMC joint with stage II and III osteoarthritis. On follow-up, three months later, they reported an improvement in the average grip and pinch strength and reduction in pain during action and rest but no statistically significant difference was identified when compared to the preoperative values. However, improvement in hand function as reported by DASH score was significantly noted. Although this study had a very small sample size and short follow-up, the same authors, three years later, published a larger case series including 50 patients with a longer follow-up period (one year). In this study, which involved patients with stages II, III and IV thumb CMCJ arthritis, intra-articular fat injection resulted in a significant reduction in pain and significant improvement in pinch and grip strength and QuickDASH score, but its main limitation was the lack of controls [17].

In 2019, Haas et al. [18] published a second study with the highest number of patients in literature so far. Ninety-nine patients with symptomatic and radiologically confirmed thumb CMCJ osteoarthritis underwent 1–2 mL of autologous fat transplantation, which had been mechanically homogenized as in their previous study [21]. The authors noticed a significant reduction in pain and a significant improvement in DASH score and MHQ, but pinch and grip strength did not change after 12 months.

Similar findings were reported in a 2-year follow-up study by Frauscher et al. [19] who injected autologous fat in 31 patients with stage II and III osteoarthritis. Finally, Bohr et al. [20] reported improvement of DASH score after intra-articular injection of fat in a patient with stage II CMCJ arthritis. However, this is a single case report and it lacks objective clinical evaluation and controls.

Among the published studies, no significant complications were reported apart from one patient who experienced hematoma after liposuction and three others reporting decreased sensitivity in the skin area innervated by the superficial radial nerve [17,18,22]. Furthermore, the routine radiological follow-up did not reveal any calcifications or further joint degeneration after fat transplantation at three months of follow-up [16]. In terms of symptomatic pain relief following fat grafting, the outcome was consistent and only four patients required revision with surgery [17,22].

## 4. Discussion

Although the number of available studies is limited, the results of intra-articular injection of autologous fat in thumb CMCJ osteoarthritis are positive and very promising. Despite the heterogeneity of patients among different studies, such as including patients with different stages of osteoarthritis (from stage I to stage IV), intra-articular injection of fat resulted in reduction in pain with subsequent improvement in hand function, as reported by DASH and MHQ scores during a variable period of follow-up (from three months to two years). In terms of grip and pinch strength, the results were conflicting and ranged from no change to significant improvement. More specifically, in three studies no improvement in pinch and/or grip strength was reported [18,19,21]. In another trial, the grip strength was only subjectively evaluated before and after fat injection and objective measurement of grip strength with dynamometer was performed only postoperatively [19]. Therefore, direct comparison was not performed and the overall benefit could not be estimated. In general, all the published studies proved the safety of fat grafting and the absence of major complications. Moreover, and when compared to resection arthroplasty, the technique was associated with a shorter operating time [22].

The fat processing technique, which is a very important step to reduce contaminants within the lipoaspirate—such as fluid, blood cell fragments and oil–and preserve adipocytes and stem cells, has been characterized by heterogeneity among the reviewed studies. There are three main types of fat processing that involve centrifugation, gravity separation and washing [24]. It was shown that washing preserves a greater number of adipose stem cells, while centrifugation can cause damage to the adipocytes and stem cells due to centrifugal forces [25]. However, results in cell yield after each fat processing technique differ and should be interpreted with caution because they vary greatly depending on the isolation protocol and the method of quantification [26]. In the reviewed articles, centrifugation was the most common method of choice, either alone or in combination with washing [17,20,22,23]. Mechanical homogenization, which was also frequently used, is a method of tissue fragmentation that can reduce the size of fat particles up to 400–600 μm. When it is processed by sequential passes through different Luer lock sizes, it gives a product known as nanofat.

The latter is rich in regenerative stromal cells and lacks mature adipocytes, which have been ruptured due to the shear forces [27]. In the studies where the lipoaspirate was mechanically homogenized, no sufficient data were reported regarding the size of the fragmented tissue and the content of the final product in viable cells [18,19,21].

In several human studies injection of mechanically or enzymatically isolated adipose-derived cellular products into knee joints with osteoarthritis can give promising results in terms of symptomatic relief of pain and improvement in joint function [6,7,28]. However, despite numerous studies investigating the effectiveness of adipose-derived products in knee osteoarthritis, there are limited data regarding their regenerative effect and clinical application on small joint arthritis, like thumb CMCJ arthritis. In a published study by Mayoly et al. [29], a mixture of fat and platelet-rich plasma (PRP) was developed as an advanced therapy medicinal product and it was injected intra-articularly in three patients suffering from grade 4 radiocarpal arthritis. The fat was harvested with a small cannula with holes of 1 mm and it was mixed with PRP. Three months following injection and up to one year thereafter, all patients reported a decrease in pain and improvement in hand function, but no significant improvement in grip strength and wrist motion was recorded.

In the reviewed articles, a symptomatic improvement was observed in all patients following fat injection but the mechanism of action of the injected cells is unknown.

Theories include a cushioning effect, due to the interposed adipose tissue, or a regenerative potential, due to the presence of stem and other progenitor cells within the fat. For this reason, randomized controlled trials comparing fat grafting with nanofat or SVF are imperative to discover the main mechanism of action. However, a double effect cannot be excluded. Strong evidence suggests that the regenerative capacity of these cells is based on their paracrine action and their ability to secrete active biomolecules, such as cytokines and growth factors. Prantl at al. [30] studied the “secretome” capacity of these cells following mechanical processing of the lipoaspirate, such as centrifugation and homogenization, and reported no significant change in secretome composition when compared to the unprocessed fat. Through this paracrine action, adipose regenerative cells have demonstrated angiogenic, anti-inflammatory and immunomodulatory capacity and are considered as a promising treatment for osteoarthritis [31]. Recently, the interest has been focused on adipose-derived exosomes, which are extracellular vesicles secreted by adipose stem cells and contain bioactive molecules, such as proteins, microRNAs, IncRNAs and mRNAs [32]. In one study, exosomes derived from adipose stem cells were shown to downregulate inflammation and oxidative stress and mediate antisenescence effects in osteoblasts obtained from patients’ knees with severe osteoarthritis [33].

## 5. Conclusions

Adipose cell-based therapies have attracted increasing attention due to their unique bioactivities and potential in regenerative medicine and their use may be applied in patients suffering from degenerative disorders, including osteoarthritis of small joints.

Based on this review, intra-articular injection of autologous fat can reduce pain and improve hand function in patients with thumb CMCJ arthritis. However, future studies should focus on identifying the optimal fat processing technique that will improve the biological properties and efficacy of the injected cells. Moreover, randomized controlled trials are necessary to assess whether injection of adipose-derived products could play a noticeable role in the symptomatic treatment of thumb CMCJ arthritis and prevent or delay the necessity of a major reconstructive procedure.

## Data Availability

Not applicable.
